# Current Healthcare Systems in Light of Hyperendemic NCDs and the COVID-19 Pandemic: Time to Change

**DOI:** 10.3390/healthcare11101382

**Published:** 2023-05-10

**Authors:** Abdelbaset Buhmeida, Mourad Assidi, Bruce Budowle

**Affiliations:** 1 Center of Excellence in Genomic Medicine Research, King Abdulaziz University, Jeddah 21589, Saudi Arabia; 2 Department of Forensic Medicine, University of Helsinki, Universitetsgatan 2, 00100 Helsinki, Finland; 3 Forensic Science Institute, Radford University, Radford, 24142 VA, USA

**Keywords:** healthcare system, noncommunicable diseases, hyperendemic, health disparities, planetary health, health determinants, well-being, wellness

## Abstract

Despite the significant achievements of current healthcare systems (CHCSs) in curing or treating several acute conditions, there has been far less success coping with noncommunicable diseases (NCDs), which have complex roots and nonconventional transmission vectors. Owing to the impact of the invisible hyperendemic NCDs and the COVID-19 pandemic, the limitations of CHCSs have been exposed. In contrast, the advent of omics-based technologies and big data science has raised global hope of curing or treating NCDs and improving overall healthcare outcomes. However, challenges related to their use and effectiveness must be addressed. Additionally, while such advancements intend to improve quality of life, they can also contribute the ever-increasing health disparity among vulnerable populations, such as low/middle-income populations, poorly educated people, gender-based violence victims, and minority and indigenous peoples, to name a few. Among five health determinants, the contribution of medical care to individual health does not exceed 11%. Therefore, it is time to implement a new well-being-oriented system complementary or parallel to CHCSs that incorporates all five health determinants to tackle NCDs and unforeseen diseases of the future, as well as to promote cost-effective, accessible, and sustainable healthy lifestyle choices that can reduce the current level of healthcare inequity.

## 1. Introduction

The structure and function of the healthcare system are deeply rooted in key discoveries and innovations, primarily developed in late 1800s and early 1900s. These breakthroughs and advancements, although diversified, time-dispersed, and, at times, seemingly unrelated, have been harmonized to contribute to the tools and practice of current medical care. Current healthcare systems (CHCSs) are grounded in more than a century-long history of technological and application achievements that have cured and/or provided treatment for a number of acute conditions that have benefited health care and contributed to improved quality of life. In this review, first, we revisit some of those accomplishments to recognize the groundbreaking contributions that have been made. The current healthcare system approach has made significant strides and, thus, continues to be supported as the most appropriate approach to address all diseases. Second, we point out that the current system has substantial limitations in light of today’s healthcare needs and has been far less successful in addressing noncommunicable diseases (NCDs). Third, we propose that a well-being-oriented system should be implemented that complements CHCSs but, importantly, incorporates the five health determinant categories to cure and/or treat NCDs. Omics-based technologies and big data science solutions are notable and are touted to improve health care. However, these solutions should not be seen as a siloed solution, as they do not adequately address a comprehensive healthcare approach. A healthcare system must promote sustainable healthy lifestyle choices, be accessible to all, and be cost-effective to address the limitations of current healthcare systems and mitigate inequities. There is a need to effect a better overall healthcare system that improves quality of life and meets the need of the greater population while not exacerbating the disparities and inequities among vulnerable populations. This review and proposal for a well-being-oriented healthcare system is based on data and experiences from Westernized systems; however, the philosophy and concepts should apply worldwide to make strides in providing equitable and accessible healthcare and promoting advancements to address NCDs.

## 2. The Bright Side of Current Healthcare Systems

Abraham Flexner is considered the father of contemporary medical education, laying the foundations for evidence-based medicine [1,2]. Additionally, innovations in healthcare quality and hygiene implemented by Florence Nightingale [3] and significant discoveries in laboratory medicine, microbiology, and imaging technologies have shaped contemporary healthcare systems. While the discovery of vaccination is attributed to Louis Pasteur [4] and von Behring [5], the modern antibiotics era ushered in the “magic bullet” concept proffered by Paul Ehrlich [6]. This notional concept was reinforced by the serendipitous discovery of penicillin—the revolutionary “wonder drug”—by Alexander Fleming [7] that selectively kills pathogenic microbes but not the host [8]. Furthermore, the systematic screening approach proposed by Ehrlich became the gold standard of drug search strategies within the pharmaceutical industry, resulting in the identification of thousands of medications for clinical use [8]. Health care was strengthened substantially by sanitation and sterilization processes promulgated by sanitary commissions led by Clara Barton during the American Civil War [9] and by Charles Chamberland, who was instrumental in the development of the autoclave [10,11].

Advances in biotechnology in the 20th century had a paramount impact, shaping modern healthcare and pharmaceuticals through the development of several technologies and products, including genetically engineered proteins, monoclonal antibodies, cell culture, polymer science, gene editing, molecularly engineered vaccines, etc. [12].

Century-long innovations and discoveries [13] have shaped CHCSs. One of the goals of CHCSs is to provide prophylaxis, such as through vaccination, but their prime mission is to be a frontline barrier against acute diseases and a provider of reactive treatment for people to live longer and healthier. Scientists and clinicians worldwide have made laudable efforts to introduce varied but impactful innovations to enhance healthcare services and alleviate the burden of human diseases. Undoubtedly, healthcare-related technologies (biotechnologies and devices), as well as clinical medicine, have improved lives and shaped today’s world. Furthermore, vaccines and antibiotics have prevented the deadly impact of most of the challenging communicable diseases. Today, there are vaccines to immunize against more than 20 life-threatening infectious diseases, such as influenza, pneumonia, diphtheria, polio, pertussis, measles, etc. As many as 3.5–5 million deaths are prevented each year due to immunization [14]. Prior to the discovery of antibiotics, infectious diseases accounted for most of the morbidity and mortality worldwide. Antibiotics revolutionized the treatment of infectious diseases worldwide, although disproportionately in developed countries, and have contributed to increasing the average life expectancy, for example, in the USA, from 47 years to 78.8 years in the twentieth century [15]. However, these benefits would not have been possible without instituting commensurate major environmental and social infrastructure, such as the wide availability of potable water, sewage services, food supply, and nutrition education programs.

Another crucial innovation was the discovery of anesthetics and antiseptics, allowing for sterile surgical procedures without pain. Opiates and herbal concoctions have been used for millennia, but it was William Morton, a dentist, who, in the mid-nineteenth century, showed the world that surgeries could be performed without pain to the patient by inhalation of diethyl ether [16]. Joseph Lister is credited with ushering in antiseptic surgical practices [17]. Based on the research of Pasteur, Lister became astutely aware that septic properties were due to microbes and employed carbolic acid to destroy “the septic germs” that were causing the decomposition of injured parts of compound fractures. The discovery of X-rays by Wilhelm Röntgen [18] and subsequent imaging innovations revolutionized disease assessment and diagnosis. These innovations contributed to the capability of performing increasingly complex surgical procedures, such as organ transplants and gynecological interventions, leading to longer life expectancy and more successful pregnancy and delivery rates.

Research on monoclonal antibodies, vector-driven mechanisms, nanotechnology, and genetic engineering has promoted effective point-of-care theranostics, thereby reducing undesired biological effects. In addition to recombinant biopharmaceuticals (e.g., insulin), DNA sequencing has contributed significantly to the diagnosis of inherited and communicable diseases, tracking of foodborne disease, parentage testing, and forensic genetics, to name a few applications. Additionally, evidence-based medicine has taken a huge step forward, exploiting clinical epidemiology [19] and randomized controlled trials [20].

## 3. Epidemiologic Transition and the Emergence of Healthcare Challenges

**Figure 1:**
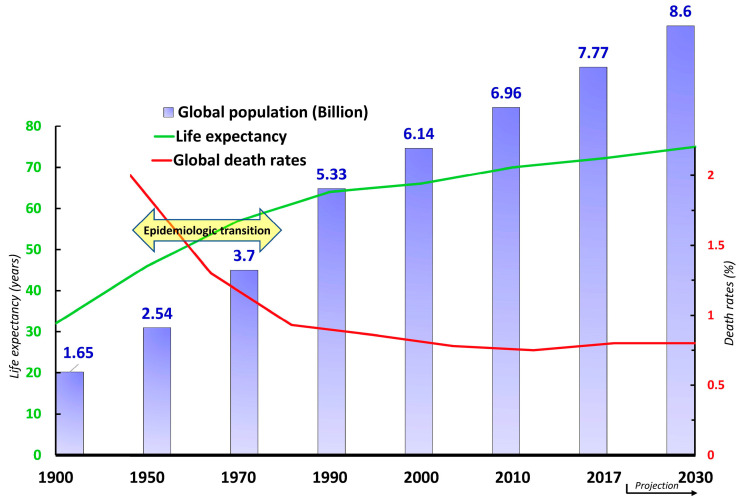
Illustration of the epidemiologic transition in the mid-20th century. **Purple**: global population growth from 1900 to 2017. Based on 2030 projections, the world population is expected to reach 8.6 billion, five times greater than in 1900. **Green**: average life expectancy (in years) increased by 2.25 times between 1900 and 2017 and is projected to reach 75 years by 2030 [29]. **Red**: global death rates (%) significantly declined from 2% in 1950 to 0.75% in 2017 [30].

These innovations have increased average life expectancy and shaped the world population pyramids/dynamics towards an important epidemiologic transition [21]. This transition is the process by which the patterns of mortality and diseases are shifting (although still persisting with notable disparities) away from high infant mortality, malnutrition, and dominance of infectious diseases affecting all age groups. In turn, health concerns are focused on aging populations, a predominantly engineered environment, and the emergence of noncommunicable diseases (NCDs) [22,23] (Figure 1). While CHCSs were initially designed to tackle acute problems, mainly infectious diseases, they have not had similar successes with NCDs and struggle to treat, cure, and/or eradicate them. Moreover, they do not reach all people and populations (and their subgroups) equally; thus, they contribute to greater health disparity as opposed to increasing health and wellness for all.

Assidi et al. [24] reported that cost prohibitive treatments tend to serve a small and often privileged sector of society and only improve quality of life for a few. Concomitantly, these treatments contribute to an ever-increasing health disparity among vulnerable populations, such as low- and middle-income populations, poorly educated people, gender-based violence victims, and minority and indigenous peoples, to name a few. Socioeconomic vectors; critically important factors such as economic growth, urbanization, aging, education, and globalization; and the pervasiveness of unhealthy products on the market need to be better supported as an important part of comprehensive healthcare strategies.

According to the World Health Organization, health equity is “the absence of unfair and avoidable or remediable differences in health among population groups defined socially, economically, demographically or geographically” [25]. For all people to achieve health equity, regardless of societal constructs, ethnicity, religion, gender, etc., they should have the same access, prospects, and resources to reach their highest health potential (Health Equity Leadership and Exchange Network [26]). Moreover, health disparities should be considered preventable differences in the burden of disease, injury, and violence experienced by the vulnerable and socially disadvantaged [27,28]. The healthcare systems of all societies should include strategies and policies in their programs to reduce disparity, with a major portion dedicated to promoting sustainable healthy lifestyle choices.

## 4. The Invisible Hyperendemic NCDs

**Figure 2:**
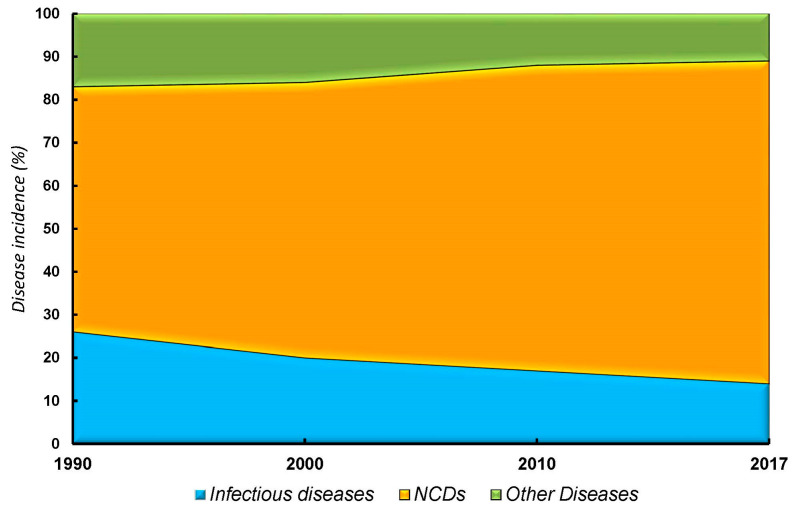
Spectrum (%) of the three (3) main global death causes from 1990 to 2017. **Blue**: decrease in the burden of infectious diseases from 21% in 1990 to 14% in 2017. **Orange**: steady surge of the global burden of NCDs. The prevalence of NCDs has increased by nearly 13% from 1990 to 2017. **Green**: the other causes of death category encompass trauma, natural disasters, crimes, terrorism, and nutritional deficiencies, among others. This category showed a trivial reduction of about 0.6 % [32,33]. Obviously, there was an uptick in infectious disease from late 2019 through 2022, but these general trends likely still hold.

For the majority of the 20th century, infectious diseases, e.g., smallpox, polio, measles, etc., were the main health concerns. However, with a reduction in the prevalence of such diseases, the epidemiologic transition has uncovered the pre-existing and likely upsurge of NCDs, which have become the major current health concern (excluding the recent COVID-19 pandemic) (Figure 2). Cardiovascular diseases, cancer, chronic obstructive pulmonary diseases, type 2 diabetes mellitus, stroke, and mental illnesses [31] were not among the top-five most common diseases in the early 1900s.

Therefore, CHCSs are just beginning to address NCDs (Figure 2) and have failed to eradicate or manage them as effectively as the deadly viral pandemics that have plagued humankind for centuries. Estimations predict that the global budget burden of NCDs will reach around USD 47 trillion by 2030 [34]. NCDs are responsible for ~71% of deaths worldwide, a figure that is projected to reach 75% by 2030 [35]. In Europe, NCDs are responsible for 86% of deaths and 77% of disease burden [36]. Beyond the threat of overwhelming CHCSs [37,38], NCDs distress individuals, families, and communities in developed and developing countries as a result of their prevalence, comorbidities, stigmatization, imposition of workforce disability, economic burden, and as causes of premature death. Additionally, the global spread and chronic nature of NCDs have led to recurrent medical interventions, lost productivity, elevated healthcare expenditures, and hampered economic development [31,39,40].

These pandemic-like features and similarities with infectious conditions may categorize NCDs as a “hyperendemic” [31,39]. Since the genetic predispositions, expression, and transmission vectors of NCDs are embedded in unhealthy modern lifestyles (mainly sedentary behavior and unhealthy dietary patterns) and predominantly engineered environments [41,42,43,44], this classification has not yet been adopted and remains controversial.

CHCSs also played an essential role in the transformation of previously life-threatening acute diseases into chronic diseases, which has allowed patients to live longer but under comprehensive and continuously monitored care. This approach to treatment is currently valid for both infectious diseases and NCDs [45]. Except for the successful eradication of smallpox and polio (nearly eradicated) [46,47], many infectious diseases continue to be a burden on CHCSs, in part due to healthcare disparities and inequities.

Overwhelmed by the challenges of managing, treating, curing, and/or eradicating NCDs, CHCSs require transformative changes to mitigate their global burden and flatten their ever-increasing curves. Since people aged over 65 years are most vulnerable, Fries proposed the compression of morbidity hypothesis, according to which NCDs could be postponed to a shorter period before death mainly through lifestyle changes [48]. This compression of comorbidities model could reduce the burden of NCDs, reduce disability-adjusted life years, and enhance overall quality of life [49,50]. However, the failure of this model to be validated and achieve profitability has impeded its implementation as part of enhanced public health promotion strategies [38,51]. The challenge of motivating individuals to change lifestyles is another impediment that requires substantial education and incentivization to effect such compression.

## 5. The Fragmented “Illness Care” System

Overwhelmed by the challenges of managing care, the essence of the CHCS model is a reactive approach whereby the system waits until a person becomes ill with acute symptoms to undertake corrective action. Even with some preventive well-being, primarily ill individuals with acute conditions may benefit from this approach, while healthy individuals may not. Thus, this acute care paradigm could be described as an “illness care” system. The mission of CHCSs tends to focus on disease management and symptom relief as opposed to health maintenance and promotion [52]. The interaction between CHCSs and individuals mimics the “swimming pool” model, in which a lifeguard does not teach swimmers the required skills but instead keeps an eye open and intervenes only when one is at risk of drowning. However, teaching people necessary preventive measures and incentivizing them to adhere to health practices is more cost-effective and valuable to public safety than primarily relying on a reactive approach.

Despite the achievements of this reactive system approach in curing several acute conditions, e.g., injuries, life-threatening circumstances, bacterial infections, and organ failure, it has struggled to cope with NCDs, the current major health and economic burden. In fact, the reactive approach of CHCSs towards managing hyperendemic NCDs is inadequate due to new, complex, and poorly identified factors embedded in global Westernization, as well as insufficient preventive strategies [52,53]. To be more effective, changes are required to mitigate the global burden of NCDs and reduce their incidence.

Since their implementation, several structural, technological, and economic aspects have been embedded inside CHCSs to enhance their effectiveness against acute conditions. Medical and technological breakthroughs provide powerful tools for pathomolecular understanding and management of diseases. Medicine has long been a laboratory/technology-dependent practice. Due to rapid scientific progress, the medical field has split into even more specialized fields/subfields [54]. Since the 1980s, the number of medical subspecialties has jumped from 40 to more than 120, and additional technology-related specialties are expected to emerge [55]. These “microspecialties” require expertise, dedicated technology, optimized investigation protocols, and properly trained staff. However, as medical systems have become more specialized, coordination between subspecialties has become more challenging [45,56,57]. Therefore, healthcare complexity can be cumbersome when trying to use recommendations from each subspecialty to formulate comprehensive medical interventions that are holistically fit to the patient [58].

Because of the palliative approach of CHCSs when dealing with NCDs, hospitals and clinics are overwhelmed with patients who have been repeatedly scheduled for clinical follow-ups. The absence of an effective cure for NCDs and their comorbidities has increased the pressure on CHCSs in terms of crowdedness, high inpatient bed demand, and long waiting lists [59]. Nowadays, hospitals are often seen as crammed shopping malls where patients are searching desperately for their health and well-being. Under CHCSs, patients are shuttled/ping-ponged among subspecialties, departments, and hospitals with little or no coordination and oversight [42]. This “revolving door” phenomenon with frequent patient in-and-out cycles fails to fulfill patients’ expectations and needs to deliver effective treatments for preventable NCDs or at least to “compress” the effects [60].

Bed capacity is still considered a key performance indicator (KPI) of hospitals in a reactive approach. This metric can be misleading, since it only alleviates the acute exacerbations of NCDs and masks their real burden (the squeaky wheel gets greased). Alternatively, in an effective proactive health system that focuses on NCDs known to be preventable, should we reconsider keeping this metric as a KPI rather than increasing it?

The deep fragmentation of CHCSs has amplified the challenges of a health system that has strongly emphasized reductionism. In fact, biomedical research, which fuels evidence-based medicine, has been historically reductionist in its understanding of human diseases by analyzing constituent cells, tissues, and organs separately. This reductionist paradigm focuses on several isolated health problems and often suggests immediate and simplistic solutions. Empowered by some achievements in treating acute diseases with known causes, this model was misemployed to treat NCDs [38]. Given their complexity, NCDs require consideration of the overall system instead of its components separately. A holistic paradigm establishes that a patient should be perceived wholly composed of interconnected and interdependent physiological, psychological, intellectual, and spiritual dimensions that collectively impact the person’s health [61]. However, both paradigms have their achievements and limitations and are complementary in delivering comprehensive and effective health care.

## 6. Healthcare Commodification

In an attempt to enhance management of CHCSs, policy makers and medical governance officers have contributed by specifying the duties, costs, and incentives of each hospitalization, including inpatient/outpatient medical services, laboratory tests, and treatment [58]. However, the gap between benefits and paid costs is widening. The low value and high price of healthcare services have motivated individuals to overcome uncertainties by self-adopting preventive approaches regarding health and well-being. Therefore, the market has become flooded with health-related commodities, e.g., healthy foods, supplements, gym memberships, cosmetic, and specialized surgeries [62,63]. Consumerism has initiated a privatization trend that has transformed a portion of CHCSs into a for-profit industry. This trend of health commodification compromises the values and healing occupation of the healthcare sector and deeply affects the authentic trustful relationship between doctors and patients [64]. Thus, health care has become service/benefit-oriented rather than value-oriented, which substantially contributes to health disparities. Unfortunately, the cost-efficient, for-profit approach of CHCSs has unintentionally generated an ethically unsustainable context that transforms caregivers into healthcare employees (i.e., deprofessionalization) and patients into customers (i.e., consumerization) [65,66]. This deeply “dehumanized” healthcare approach has incited poor patient satisfaction and worsened the inequality of healthcare distribution and accessibility. Taken together, there is a clear need for a transformative change of CHCSs [38,67].

## 7. Skyrocketing Healthcare Expenditure

The fragmentation and commodification of CHCSs have led to managerial and financial burdens characterized by growing human resource staffs, bureaucracy, and fragmented services. Health expenditure in developed economies is increasing more rapidly than the gross domestic product (GDP) [68]. In the USA, health cost per capita is projected to rise from 15% of the GDP in 2005 to 25% and 37% in 2030 and 2050, respectively [68,69,70]. Economists estimate that the unprecedented expansion and application of specialized medical technologies are among the main cost drivers, contributing to 40 to 50% of the USA’s annual healthcare cost increase [71]. The appropriate use of these technologies (avoiding unnecessary/overlapping tests) can be a key factor to control rising costs. The increase in health costs has also increased personal out-of-pocket healthcare expenditures. Poorer quality of services at higher costs have fostered mistrust towards CHCSs and promoted options such as alternative medicine [65,72].

Additionally, the biomedical technology revolution has created a gap between medical curricula, healthcare practitioners, patients, and companion theranostics [38]. Extra costs related to curricular updates, advanced training, drug discovery, technology maintenance, and patient awareness also need to be counted [73].

Recent studies report a massive waste of resources within CHCSs (20–40%) that makes cost-reduction and value-enhancing efforts even more challenging but absolutely necessary. The WHO estimates that the lack of efficiency includes tests overutilization, unneeded interventions, unintended medical errors, unnecessary lengthy hospital stays, unsustainable costs, and harm to patients [74,75,76,77]. In Europe, elderly NCD patients were found to concurrently receive fragmented, repeated, and poorly coordinated care from several specialists and departments (41). These skyrocketing costs are unaffordable for many people and are signs of the unsteadiness of CHCSs. Such dramatic rises in healthcare costs without equivalent rises in quality are alarm bells for the urgency of necessary change.

## 8. Unfilled Quality Gap

Medical errors are recognized as the third leading cause of death in the USA [78], despite the challenges associated with recording such events. This healthcare limitation has fostered mistrust and pressure on CHCSs to make substantial improvements to service quality, availability, affordability, accessibility, safety, and cost-effectiveness. Consequently, international committees and experts have suggested strategies/initiatives for quality improvement. These efforts are aimed to bridge the gap between CHCS quality and public expectations by developing equitable and timely patient-centered services that are individualized, effective, safe, and efficient [79,80].

The focus of quality improvement initiatives is to define how to assess and improve essential aspects of healthcare provision, such as standardization, accreditation, professional qualifications, health technologies, auditing, safety, patient involvement, and cost-effectiveness. Frameworks for quality initiatives include “To err is human: Building a safer health system” [81], “Crossing the Quality Chasm” [79], “The Innovative Care for Chronic Conditions (ICCC) framework” [82], “Unintended Harm in Medical Practice” [83], “Choose Wisely Campaign” [84], and “The Goal of Zero Harm Campaign” [85], to name a few. However, most of these initiatives have been slow to implement, difficult to expand, and rarely adopted on a large scale.

The formidable gap between biomedical research and healthcare delivery also needs to be addressed [86,87]. A noticeable and sustainable improvement in the provision of high-quality health care requires more than simple, short-term solutions. Furthermore, experts estimate that the contribution of medical care as a determinant of individuals’ health in reducing premature death will not exceed 15%, regardless of the level of quality improvement in CHCSs [88].

Fundamentally, overstressing CHCSs will not deliver tangible solutions regarding the gap between healthcare quality and public expectations. These issues are not due to a lack of goodwill, dedication, or know-how but rather due to intrinsic inadequacies in CHCSs [79]. Shortcomings in terms of environment, processes, capabilities, and scalability will make the achievement of high quality extremely challenging; perhaps building a new system should be considered.

## 9. CHCS Shortcomings in Response to the COVID-19 Pandemic

Sadly, the COVID-19 pandemic has, as of 12 April 2023, affected 762,791,152 people, of whom 6,897,025 have died [89]. This pandemic has further exposed the deep crisis of CHCSs and their incapacity to handle COVID-19 patients with severe respiratory symptoms. Without taking into account instances of unethical denial of care for non-COVID-19 patients, the unavailability, inaccessibility, inequality, and ineffectiveness of CHCSs have been noticeable [90]. The inadequacy of CHCSs has been evidenced by a severe lack of personal protective equipment, intensive care unit beds, and ventilators to align with the excessive demand, a situation which has led to unprecedented frontline healthcare provider fatalities [91,92]. CHCS infrastructure is not prepared for surge capacity, which is exacerbated by crises such as wars and political instability.

The public healthcare crisis has been aggravated by concomitant economic and humanitarian crises [93]. Due to the lack of effective emergency management plans in CHCSs, NCD patients have been unethically left to fend for themselves, forced to choose between postponing their medical check-ups with a risk of fatal exacerbations or taking the risk of contracting the virus or other nosocomial microorganisms during their hospital visits due to some being already immune compromised.

Compounded by an increase in COVID-19 cases, unprepared CHCSs, and the absence of a treatment or vaccine, governments immediately sought to restrict social and lifestyle choices that would alleviate the burden of the pandemic until herd immunity was reached. Lockdown, isolation of confirmed cases, quarantine of suspects, and social distancing have been among the social measures implemented by governments. These actions, together with lifestyle commitments such as using face masks, frequent hand washing, healthy diet, exercise, and adequate sleep, are considered key tools to reduce the spread of the virus and flatten the epidemiological curve (simpler solutions matter) [24]. Without these social and lifestyle measures that were historically marginalized by CHCSs, the COVID-19 pandemic could have been a population-threatening disaster if it had not been for vaccine development and large-scale production [94].

## 10. Additional Challenges of CHCSs

The crowdedness of CHCS clinics and the surging number of disabilities and premature deaths worldwide manifest the failure of a fragmented “sickcare” system (functional sickness) unable to manage NCDs. Moreover, CHCSs are facing a deep structural crisis (structural sickness) beyond all reform and quality initiatives. This “double-sick system” (twofold failure at both structural and functional levels) cannot fulfill patients’ needs. Thus, changing (or at least modifying) how CHCSs function and how they are structured should be considered.

However, some healthcare stakeholders have been resistant to change what they perceive to be a functional care delivery system [95]. Their ability to override change at different levels hampers potential reform of CHCSs. These stakeholders should be held accountable for keeping the medical curricula and healthcare professionals deeply submerged in the disease while marginalizing prevention and public health promotion [52,53].

Remarkably, 97% of biomedical research funds in the USA are allocated to treatment (disease-focused), with only 3% directed to prevention and health promotion (wellness-oriented) [96]. Despite these investments, CHCSs continue to face a treatment crisis rather than a diagnostic crisis [97]. No single treatment has been proven effective in eradicating any NCD to date, except some of their symptoms/exacerbations, which does not stop disease progression [52].

## 11. The Promise of Health-Determinant-Oriented Care

To demystify the limitations of CHCSs, the main determinants of human health should be addressed. A health determinant is a main causal factor (or a group of interconnected factors) that decisively influences the health and well-being of individuals or populations. Individual health is a state of homeostasis resulting from the interaction of several intrinsic (e.g., genome, biology, and psychology) and extrinsic conditions (e.g., environmental, social, cultural, and economic contexts). Extrinsic conditions are those that, if addressed appropriately and cost-effectively, may more efficiently contribute to reducing disease burden, reinforcing natural systems, enhancing eco-friendly lifestyles (promoting physical activity and active mobility, reducing sedentary behavior, strengthening sustainable food systems, etc.), tailoring customized community empowerment programs, and promoting achievement of optimal health for the wider population.

**Figure 3:**
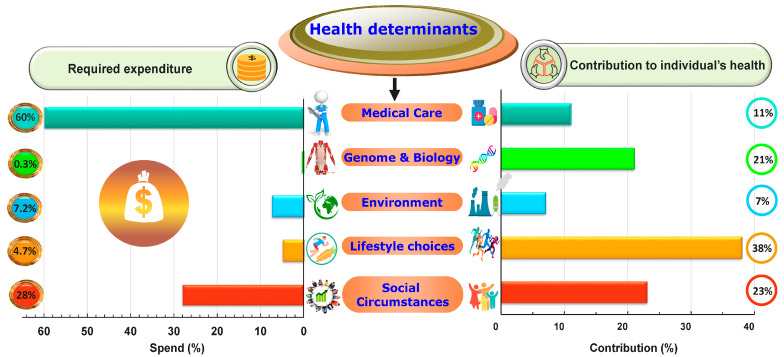
Relative contributions of the five health determinants (%) and their corresponding required expenditure (% of total expenditure). **Center**: the five determinants of health. These five domains interact with one another to determine human health (see reference [105], accessed on 20 January 2022). **Right**: relative contribution of each determinant to individual health (%). Each health determinant has a specific relative weight/importance. **Left**: required relative expenditure (%) dedicated to each health determinant. Expense proportions were estimated from a preliminary cost analysis study conducted in the USA. Lifestyle choices and social circumstances make up 61% of individual health—about six times the contribution of medical care at half the cost. Note: These expense estimations are approximations and may vary over time and among countries.

Several studies have shown that there are five main health determinants: (1) genome and biology, (2) lifestyle choices, (3) social circumstances, (4) physical environment, and (5) medical care [88,98,99,100,101,102,103,104,105,106,107] (Figure 3). Social (23%) and lifestyle (38%) determinants make up 61% of individual health.

Social determinants constitute the socioeconomic context in which people live [108] and encompass income, education, discrimination experiences, employment, marital status, neighborhood, and social networks. Lifestyle choice determinants include smoking, dietary habits, sleeping patterns, physical activity, mood levels, and beliefs. Genome and biology determinants include overall susceptibility, biological and molecular cross networks, and body homeostasis; these elements determine 21% of individual health. Additionally, the physical environment makes up 7% of individual health and well-being and includes housing conditions, location, transportation, safety, entertainment, ecology, and quality of water and air. Strikingly, the contribution of medical care to individual health is only 11%; it encompasses the structure, governance, accessibility, quality, affordability, and outcomes of CHCSs. Thus, 89% of an individual’s health is determined by factors outside of the healthcare realm, i.e., genetics/biology, behavior, environment, and social circumstances.

The identification of health determinants is the result of studies that have integrated, curated, and visualized their relative weights of importance [88,107,109]. Given their complexity of interactions, the estimated relative weight of each health determinant to individual health is dynamic and depends on an array of cross-interacting factors (e.g., magnitude, space, and time for each factor) during the human life course [88,110]. Consequently, several experts, commissions, and non-governmental organizations have overwhelmingly recognized the limitations of CHCSs and have called for immediate and deep health-determinant-oriented reform [36,38,52,111,112,113].

A preliminary cost-effectiveness analysis of the expenses related to different health determinants was mapped in the USA in 2018 using federal, state, and private expenditure data. Of the total budget allocated to health determinants (about USD 5.58 trillion), the spending proportions of medical care, social circumstances, physical environment, lifestyle choices, and biology were 59.8%, 28%, 7.2%, 4.7%, and 0.3%, respectively (Figure 3) [109]. The contribution to individual health of both social and lifestyle choice determinants (61%) is about six times greater than that of medical care (11%) (Figure 3). Surprisingly, medical care expense is twice that of social and lifestyle determinants combined (Figure 3). Although these spending estimations may vary over time and among regions, it is evident that investment on socioeconomic and lifestyle choices should increase and could be more effective and far less costly than continuing with the current CHCS paradigm.

Tangible reform and reduction in health disparities will not occur unless the role of different health determinants is emphasized and addressed.

## 12. Conclusion and Perspectives

In this review, the ineffectiveness of CHCSs, mainly against NCDs, the main health threat of the 21st century, is raised. Although CHCSs are still effective in dealing with acute conditions (such as bacterial infections, tropical diseases, and mechanical injuries), they are not adequately adapted to tackle NCDs and are more focused on the management of their severe exacerbations. Quality initiatives to combat the structural and functional issues of CHCSs are still unable to bridge the quality gap. The meager approach of CHCSs towards NCDs is unlikely to flatten their uprising curves.

Ignoring or marginally contributing to other health determinants has substantially hindered the performance and growth and CHCSs. The marginalization of prevention and health promotion—partly due to unprofitability—has impeded proper implementation of health quality pillars and adequate prevention strategies. In the digital era, innovations such as omics technologies, connected health systems, wireless wearable devices, blockchain technology, the Internet of Things, health tokens, artificial intelligence, and machine learning are promising ways to address the challenges of CHCSs [97,114,115]. However, serious challenges with respect to their implementation, costs, and possible outcomes are foreseeable and understandable. It is likely that the current “magic bullet” illusion in CHCSs will eventually be exacerbated by another upcoming “omics and big data” illusion.

The complexity of human biology, lifestyle choices, and their dynamic (time and space) interactions with longer exposure (due to longer life expectancy) to the socioeconomic and ecospheric environment remain underestimated, marginalized, and poorly investigated. Some CHCS reform efforts may not be effective in tackling hyperendemic NCDs. Thus, the billion/trillion-dollar question is: Will society maintain a fragmented and ineffective CHCS or seriously develop a new and effective NCD-oriented care system?

From our point of view, CHCSs should be dedicated to dealing with acute diseases (infections, injuries, and exacerbations of NCDs) (acute agenda). However, in parallel, a complementary system should be developed that incorporates the remaining health determinants with goals to tackle NCDs (chronic agenda) and address the unforeseen diseases of the future (i.e., a futuristic agenda). The stakeholders in this new system should be health-oriented rather than disease-oriented, investing in sustainable wellness (salutogenesis) rather than disease (pathogenesis). A comprehensive strategy that incorporates all health determinants to globally impact all individuals and communities should include political commitment, leadership, and governance; universalism and environmental friendliness as core values; engagement of stakeholders and private sector providers; active community involvement and wellness-driven solutions that include training, education, and outreach programs; community empowerment programs and policies; development, implementation, and evaluation of culturally and linguistically appropriate well-being-based initiatives; and the redesign of the healthcare and well-being blueprint to commit to all health determinants for optimal personalized and community wellness worldwide.

## Data Availability

All relevant analyzed data are contained within the published version of this review article.
